# Drivers of the third wave of COVID-19 in Zimbabwe and challenges for control: perspectives and recommendations

**DOI:** 10.11604/pamj.2021.40.46.31237

**Published:** 2021-09-17

**Authors:** Grant Murewanhema, Faith Mutsigiri-Murewanhema

**Affiliations:** 1 Unit of Obstetrics and Gynaecology, Department of Primary Health Care Sciences, Faculty of Medicine and Health Sciences, University of Zimbabwe, Harare, Zimbabwe,; 2 Department of Community Medicine, Faculty of Medicine and Health Sciences, University of Zimbabwe, Harare, Zimbabwe

**Keywords:** COVID-19, pandemic, waves, vaccination, Zimbabwe

## Abstract

Since the beginning of June 2021, Zimbabwe entered into a harsh third wave of the COVID-19 pandemic, which saw an increase in the cumulative number of cases from approximately 38,000 to 120,000 in just two months. This exponential case rise was accompanied by an increase in the absolute number of case fatalities, with a corresponding strain on the public health sector. To effectively inform public health responses, policy and strategy to deal with the current wave and prepare for further waves, we discuss the drivers and challenges of control for this current wave and future waves, and offer practical recommendations. Vaccination will be the most important public health intervention to deal with the spread, morbidity and mortality of COVID-19, therefore, efforts to fight vaccine hesitancy and build vaccine confidence and availability will be critical. Similarly, it will be important to build public health sector capacity and resilience to adequately deal with large-scale outbreaks and absorb the shock waves associated with such. Resuscitating and building the economy is an indispensable component of protecting public health. Therefore, collaborative efforts from relevant public health stakeholders, economists, politicians and other players are required to effectively coordinate the necessary responses and formulate the right policies and strategies.

## Perspective

Zimbabwe experienced its harshest wave of the COVID-19 pandemic to date from June 2021, to end of July 2021. This wave saw the highest number of cases, resulting in a tripling of the cumulative number of confirmed COVID-19 cases from an estimated 38,000 at the end of May 2021 to near 120,000 at the end of July 2021. The first wave, which settled by the end of August 2020, left the country with less than 1000 confirmed cases, having followed a very insidious course [1]. The second wave was more severe compared to the first wave, and saw an over 300% increase in case burden from December 2020 to January 2021 [2]. The case fatality rate during both waves was reported as 3.4% [1,2]. The majority of cases reported in the first wave were imported from other countries such as South Africa, Botswana and the United Kingdom, with only a small proportion attributable to local community transmission [3]. Conversely, the majority of cases in the second wave, over 90%, were attributable to different patterns of local transmission, including clustering, sporadic and widespread community transmission [2].

The drivers of transmission for the waves have also been different. The most likely drivers for the first wave included porous borders, weak surveillance at ports of entry into the country and within the country, and asymptomatic transmission as the majority of the cases were reported as asymptomatic [3]. Owing to the milder nature of the disease, more people with infection may have been missed due to lack of symptoms [4]. Due to a very strict and thoroughly enforced lockdown, fewer cases were attributable to human mobility. On the other hand, the second wave was largely driven by complacency, increased human mobility, and the more transmissible beta variant that had its origins in South Africa. Results from genomic sequencing revealed that more than 70% of incident cases in January 2020 were due to this variant, whilst greater than 90% of cases in January 2021 were attributable to this variant [5]. The newer variants have higher effective reproduction numbers, and come with changing disease epidemiology. The drivers of the pandemic are evolving as the pandemic evolves, and as the third wave settles across Africa, it is important to examine the drivers of the third wave and the challenges faced in controlling the wave. It is now becoming apparent that the severe acute respiratory syndrome coronavirus 2 (SARS-CoV-2) may remain an important respiratory pathogen in the future, with periods of low transmission and intervening harsh waves in between [6]. It is therefore important for stakeholders in public health to design preventive and control strategies proactively. This is premised upon an understanding of the factors that may be driving the waves, and the possible challenges for control. In this present paper, we therefore give perspectives on the possible drivers of transmission in the third wave and the control challenges, and offer proactive recommendations as we anticipate further waves of COVID-19 in Zimbabwe and beyond.

### Drivers of the waves

**More transmissible variants:** globally, the delta variant, first detected in India, has been detected. This variant is more easily transmissible with a higher reproduction number, shorter incubation period and more severe clinical disease. In Zimbabwe, the delta variant was reported to be responsible for 98% of the cases in the third wave in a cabinet briefing. SARS-CoV-2, like many other viruses has been undergoing frequent mutations, and the mutant strains have different transmissibility and clinical disease spectrum [6]. The strains may also reduce the effectiveness of the current vaccines and necessitate the need for repeated vaccine doses. In populations that are vaccine hesitant [7], this may result in reduced uptake of subsequent doses.

**Pandemic fatigue and human complacency:** after a prolonged period under COVID-19 control restrictions, the population is becoming fatigued and therefore more complacent to preventive strategies. Wearing of facemasks, physical distancing and frequent hand hygiene are unnatural and tiring for many. People have become tired of being confined to certain areas, are moving about more widely, and are less likely to adhere to restrictions and control measures.

**Public health sector challenges:** the public health sector in Zimbabwe, like that of many countries in sub-Saharan Africa, was largely reported as fragile at the onset of the COVID-19 pandemic [8]. Several challenges stemming from an inadequate national budget and resource allocations towards building and capacitating the public health sector have resulted in widespread shortage of medical consumables and equipment, and lack of space for admitting and treating sick COVID-19 patients [9]. This has been worsened by the continuous migration of skilled workers to greener pastures as the hyperinflationary environment erodes earnings. This has resulted in gross work force challenges during the COVID-19 pandemic, with understaffed facilities failing to cope with the increased demands. Owing to lack of insurance and competitive remuneration, healthcare workers have remained largely hesitant to work in hazardous environments. News of the death of frontline healthcare workers have worsened the situation [10]. This has resulted in patients staying at home and seeking home-based care services. The infection prevention and control conditions prevailing in these homes are largely unknown, and might possibly have fuelled infections. Unfortunately, some of the patients have also deteriorated at home, presented to hospitals with advanced disease due to lack of proper monitoring, which has worsened their prognosis.

**Surveillance challenges:** breaking the chains of SARS-CoV-2 transmission within a wave requires effecting testing, treating and isolating of confirmed cases, as well as contact tracing with quarantining of contacts of confirmed cases until the end of the incubation period [11]. Surveillance at ports of entry is critical for arresting inward migrating of residents infected with SARS-CoV-2 until they are negative, and serves to contain imported cases. Within country, active surveillance encompasses actively testing all suspected cases, treating them and tracing all their contacts, as well as sentinel surveillance through surveillance for acute respiratory infections and influenza-like illnesses. Zimbabwe faced several surveillance challenges from the onset of the pandemic. Contact tracing is often challenged by lack of human resources, fuel and communication means such as for calling and data transmission [3]. Limited laboratory testing capacity has been a challenge, as the conventional reverse transcription polymerase chain reaction (RT-PCR) testing has been very expensive and unsustainable, while alternative polymerase chain reaction (PCR) testing on GeneXpert platforms was made difficult by a global shortage of the cartridges [3]. The cheap rapid antibody tests have very low sensitivity and specificity [12], and have not been included in the national programme. Rapid antigen testing has provided a cheaper alternative, has improved the testing capacity of the country, but has largely remained inadequate.

**Transport challenges:** from the onset of restrictive measures in March 2020, the government of Zimbabwe banned all forms of public transport except those affiliated to a national passenger company [3]. The country mainly relies on road transport, there being no efficient or working public rail systems. The transport control measures helped to control flow of human traffic during the first and second waves. However, with increased human mobility and failure/refusal to comply with restrictive measures in the third wave, the available form of transport has proved to be largely inadequate. This has resulted in overcrowding at both the stations and within the locomotives, all potential SARS-CoV-2 super spreader activities. The current transport challenges need an urgent redress.

**Overcrowding:** the government introduced vaccination in February 2021 [13]. The onset of the third wave has seen people flocking to vaccination centres. Vaccine shortages and inefficiency owing to human resources shortages and fewer vaccination centres have seen people overcrowding as they scramble for the available vaccines at the running centres. Despite restricting permitted numbers to thirty, people have continued attending some funerals in large numbers. All these overcrowding events may possibly have led to the further spread of SARS-CoV-2.

**Relaxed restrictive measures:** unlike the first and second wave where restrictive measures were seriously controlled, with traffic checkpoints and random checks on human movement, and closure of market places and informal trading places [14], the current measures have largely been relaxed or not enforced fully. In places where infection control measures such as screening, hand washing and maintenance of physical distancing must be enforced, there has been limited monitoring or compliance or both.

### Challenges for control

**Difficulties with decision-making matrices:** the socioeconomic circumstances of most Zimbabweans are difficult, with the majority of the population now living in absolute poverty as defined by the World Bank. The crux of survival for the majority is informal trading in a country where the majority are unemployed and have no disposable savings. On the other hand, lockdowns have been argued to have negative socioeconomic consequences on the population [15]. This may be more severe where there is non-existent social support from the government. The excess socioeconomic problems precipitated indirectly on the populace by the COVID-19 pandemic are difficult to estimate; however, it is widely postulated that the indirect damage may have greatly exceeded the damage caused directly by the disease. Additionally, academics have questioned the value of lockdowns in countries where more people die annually from other diseases such as tuberculosis, malaria, HIV and many other public health challenges [16]. The government tried to introduce targeted lockdowns at the beginning of the third wave, with restrictions placed in areas that designated as hotspots. However, with continued spread, the government had to impose a nationwide but relaxed lockdown. The partial opening of the resort town of Victoria Falls attests to the dilemma of whether to keep the country locked down, or allow activities that bring in revenue inflow into the country as the government attempts to resuscitate a largely collapsed economy. Indeed, the government has been faced with a decision-making conundrum in the third wave much more than the previous waves, and with progression of time, decision-making is likely to become even more complicated.

**Recommendations:** policy and strategy must focus on bringing the current wave under control, but more importantly, must prepare the country against future waves that are inevitable, in terms of reducing the morbidity and mortality associated with severe COVID-19, and prepare the healthcare system to absorb any shock waves that may be brought about by harsher waves. We therefore make summarised recommendations as in Table 1.

**Table 1:** recommendations for controlling the third wave and preparing for further waves of the COVID-19 pandemic in Zimbabwe

Recommendation	Action items
Accelerate the vaccination programme to achieve herd immunity	Accelerate procurement of vaccines
	Adequately deal with vaccine hesitancy
	Build vaccine confidence
	Deal with logistical issues, and ensure wider availability and accessibility of the vaccines, prioritising the most vulnerable members of the population
Strengthen risk communication and community engagement	Continue reinforcing that all groups are susceptible to COVID-19
	Continue dealing adequately with the rumours, myths and misconceptions regarding COVID-19 and the vaccination therefore
	Continuously develop, adapt and distribute COVID-19 information, education and communication material in all the local languages
	Continue to widely disseminate the information, education and communication material on all possible media platforms
	Continuously develop strategies to deal with pandemic fatigue and complacency
Build public health sector capacity and resilience	Use period(s) of stability to address human resources and bed capacity challenges
	Adequately address healthcare workers’ insurance and remuneration concerns to avoid further brain drain
	Build more isolation and quarantine facilities for further waves
	Build up on stocks of consumables such as oxygen, medicines, personal protective equipment and other sundries
	Enhance the public health sector’s capacity to test, treat and isolate confirmed cases, conduct efficient contact tracing and quarantine contacts
Integrate COVID-19 prevention activities into mainstream public health activities	Reinforce the message that SARS-CoV-2 is likely to remain as one of the major respiratory pathogens in the future, therefore begin to integrate COVID-19 prevention messages into the main public health arena alongside other infections of public health importance
Enhance differentiated control strategies	During periods of low transmission, develop differentiated strategies that allow economic activities to go on whilst clusters of cases are contained
Work on building/resuscitating the economy and enhance social support for the vulnerable	The economy is the basis of a functional public health sector, and needs urgent resuscitation
	Enhance social support systems that prioritise the vulnerable and protect them during periods of high transmission

## Conclusion

Whilst control of the ongoing third wave is a high priority, the government of Zimbabwe must also focus on preparing for future waves, which are inevitable. To this end, various stakeholders in public health, including those in politics and economics must continue working collaboratively to devise effective policy and control strategies, premised upon understanding the drivers and challenges of SARS-CoV-2 transmissions and COVID-19 wave´s control. Local studies to build the evidence are required to effectively inform public health.
